# Crosslinking-Induced Endocytosis of Acetylcholine Receptors by Quantum Dots

**DOI:** 10.1371/journal.pone.0090187

**Published:** 2014-02-25

**Authors:** Chi Wai Lee, Hailong Zhang, Lin Geng, H. Benjamin Peng

**Affiliations:** 1 Division of Life Science, State Key Laboratory of Molecular Neuroscience, The Hong Kong University of Science & Technology, Clear Water Bay, Hong Kong, China; 2 Department of Physiology, Yong Loo Lin School of Medicine, National University of Singapore, Singapore; 3 Department of Physiology, Faculty of Medicine, The University of Hong Kong, Pok Fu Lam, Hong Kong, China; University of Sydney, Australia

## Abstract

In a majority of patients with myasthenia gravis (MG), anti-acetylcholine receptor (AChR) antibodies target postsynaptic AChR clusters and thus compromise the membrane integrity of neuromuscular junctions (NMJs) and lead to muscle weakness. Antibody-induced endocytosis of AChRs in the postsynaptic membrane represents the initial step in the pathogenesis of MG; however, the molecular mechanisms underlying AChR endocytosis remain largely unknown. Here, we developed an approach to mimic the pathogenic antibodies for inducing the crosslinking and internalization of AChRs from the postsynaptic membrane. Using biotin-α-bungarotoxin and quantum dot (QD)-streptavidin, cell-surface and internalized AChRs could be readily distinguished by comparing the size, fluorescence intensity, trajectory, and subcellular localization of the QD signals. QD-induced AChR endocytosis was mediated by clathrin-dependent and caveolin-independent mechanisms, and the trafficking of internalized AChRs in the early endosomes required the integrity of microtubule structures. Furthermore, activation of the agrin/MuSK (muscle-specific kinase) signaling pathway strongly suppressed QD-induced internalization of AChRs. Lastly, QD-induced AChR crosslinking potentiated the dispersal of aneural AChR clusters upon synaptic induction. Taken together, our results identify a novel approach to study the mechanisms of AChR trafficking upon receptor crosslinking and endocytosis, and demonstrate that agrin-MuSK signaling pathways protect against crosslinking-induced endocytosis of AChRs.

## Introduction

All muscle movements depend on the proper formation and maintenance of the peripheral synapses, the neuromuscular junctions (NMJs). The NMJ comprises a presynaptic nerve terminal, the postsynaptic muscle membrane, and perisynaptic Schwann cells [1], [2]. A prominent feature of the NMJ is the presence of a postsynaptic apparatus containing a high concentration of nicotinic acetylcholine receptors (AChRs) that ensures effective synaptic transmission between presynaptic neurons and postsynaptic muscle cells. During synaptogenesis, the nerve terminal secretes the heparan-sulfate proteoglycan agrin, which induces the local clustering of AChRs in the postsynaptic surface, a process that is mediated by the activation of the muscle-specific tyrosine kinase MuSK through the low-density lipoprotein receptor-related protein Lrp4 [3]–[5]. At the postsynaptic membrane, AChRs are clustered at a nearly crystalline density of ∼10,000 receptors/µm^2^, in contrast to the density of <10 receptors/µm^2^ at extrasynaptic sites [6]. Our previous studies showed that the actin cytoskeleton acts both passively as a molecular scaffold for AChR clustering and anchorage, and actively as a driver of the surface insertion of AChRs by means of vesicular trafficking [7], [8]. A receptor-associated protein named rapsyn immobilizes the surface AChRs on actin filaments by forming a ternary complex with α-actinin [9] and controls the turnover rate of AChRs [10]. However, the mechanisms underlying the regulation of AChR trafficking to and from the postsynaptic membrane remain largely unknown.

Aberrant changes in postsynaptic receptor trafficking have been linked to synaptic dysfunction in several neurological diseases associated with central and peripheral synapses. Myasthenia gravis (MG) is a prototypical antibody-mediated autoimmune disease that is characterized by the weakness of the skeletal muscle contractions at NMJs. Nearly 90% of MG patients have anti-AChR antibodies in their serum, which recognize a complex array of epitopes that differs between patients [11]. The pathogenic antibodies in MG bind to the extracellular domains of AChRs on the postsynaptic membrane that can be crosslinked with nearby AChR molecules [12], and this adversely modulates the trafficking of postsynaptic AChR clusters: antibody-mediated crosslinking induces AChR endocytosis and leads to the subsequent lysosomal degradation of AChRs [11], [13]. This antigenic modulation, together with complement activation [14], contributes to AChR loss at the NMJs in MG patients. Because the endocytosis of crosslinked AChRs is likely the rate-limiting step in antigenic modulation *in vitro* and *in vivo* [13], understanding the cellular and molecular mechanisms underlying the crosslinking-induced endocytosis of AChRs should lead to an enhanced understanding of the pathogenesis of MG and may suggest therapeutic interventions that can be used to treat this disease.

Quantum dots (QDs) are nanocrystal microspheres that emit bright fluorescence and are extremely photo-stable when compared with other conventional organic fluorophores such as fluorescein and rhodamine [15]. This unique feature has enabled us to perform long-term tracking of single molecules in live muscle cells: in recent studies, we have successfully used QDs to monitor the dynamic behavior of diffuse AChRs on the muscle surface [16], [17]. In this study, we exploited the unique features of QDs and the multivalency of streptavidin in its binding to biotinylated α-bungarotoxin [18] to establish a novel approach to effectively induce AChR endocytosis through crosslinking. Using this simple assay, we were able to identify 2 distinct pools (internalized and surface) of AChRs based on the size, fluorescence intensity, trajectory, and subcellular localization of the QD signals. We further demonstrated that clathrin and microtubules are essential for crosslinking-induced AChR endocytosis and endocytic trafficking. Importantly, we showed that the activation of agrin-MuSK signaling protects the diffuse AChRs against crosslinking-induced endocytosis, which provides insights into the regulation of crosslinking-induced AChR endocytosis triggered by the pathogenic antibodies in MG.

## Materials and Methods

### Reagents

Recombinant full-length chick agrin was produced as described previously [19], [20]. Recombinant heparin-binding growth associated molecule (HB-GAM) was kindly provided by Dr. Heikki Rauvala (University of Helsinki). Phenylarsine oxide, monodansylcadaverine, methyl-β-cyclodextrin, filipin III, and nocodazole were purchased from Sigma. Latrunculin A was obtained from Life Technologies. Anti-β-tubulin mouse antibody was purchased from Millipore.

### Xenopus Embryo Microinjection and Primary Muscle Cell Culture

The mRNAs encoding GFP-tagged full-length and truncated mouse MuSK and rapsyn were synthesized using an *in vitro* RNA transcription kit (mMessage mMachine; Life Technologies); the mRNAs were injected into one blastomere of 2-cell stage *Xenopus* embryos by using an oocyte injector (Nanoject, Drummond Scientific) as described previously [21]. GFP-expressing embryos were selected for preparing primary cultures. Muscle cells were dissociated using collagenase treatment from the myotomal tissues of injected embryos at Stage 19–22 and then plated on glass coverslips coated with a commercially available extracellular matrix mixture consisting of entactin-collagen IV-laminin (E-C-L, Millipore). The following cDNA constructs were used: wild-type and kinase domain-deleted mutant of MuSK, obtained from Dr. Alastair Reith (GlaxoSmithKline Pharmaceuticals), and wild-type and coiled-coil domain-deleted mutant (Δ297–331) of rapsyn, kindly provided by Dr. Jean Cartaud (Universités Paris 6 et 7). All the cDNA constructs were tagged with green fluorescent protein (GFP) at the C-terminus and subcloned into the pcDNA3.1+ vector for expression.

### Induction of AChR Endocytosis by QD Labeling

Cultured muscle cells were first labeled with 25 nM biotin-conjugated α-bungarotoxin (biotin-BTX, Invitrogen), rinsed 3 times with culture medium for 10 min each, and then labeled with 2.5 nM streptavidin-conjugated QD 655 (Invitrogen) for 10 min. After washing with culture medium, the cells were maintained at room temperature (23°C) for 1 h to allow AChR endocytosis to occur, after which live-cell imaging was performed.

To examine the subcellular localization of AChR vesicles, AChR endocytosis was induced through sequential labeling with 25 nM biotin-BTX and a saturating dose of QD 655-streptavidin (50 nM). Next, the cell membrane was marked by labeling GM1 by using 50 ng/mL biotin-conjugated cholera toxin B (biotin-CTX) and 2.5 nM QD 525-streptavidin. The labeled cells were mounted on a custom-made sealed chamber designed for live imaging.

### Immunocytochemistry

To perform immunostaining, *Xenopus* muscle cells were fixed with 4% paraformaldehyde in phosphate-buffered saline (PBS) for 15 min, rinsed 3 times with PBS, and then permeabilized with 0.1% Triton X-100 in PBS for 10 min. After blocking with 5% bovine serum albumin (BSA, Sigma) for at least 1 h, the cells were incubated with primary antibodies for 2 h and then with Alexa 488-conjugated secondary antibodies (1∶400 dilution; Life Technologies) for 45 min. After extensive washing in PBS, the coverslips were mounted on glass slides with an anti-bleaching agent (Citiflour, Ted Pella).

### Microscopy and Data Analysis

Fluorescence imaging of live and fixed cell cultures was performed on an inverted fluorescence microscope (IX70, Olympus). Digital images were acquired using a cooled CCD camera (ORCA II, Hamamatsu) that was controlled using MetaMorph software (Molecular Devices). Time-lapse images of AChR vesicles were captured at an interval of 500 ms for 3 min or 3 s for 30 min under the control of an optical filter changer (Lambda 10-2; Sutter Instrument). Data were analyzed using ImageJ (National Institute of Health) and MetaMorph software (Molecular Devices). Data are shown as mean ± SEM and the statistical significance of differences between the control and experimental groups was assessed using Student’s *t* test.

### Ethics Statement

All animal experiments in this study were carried out in strict accordance with the protocol approved by the Hong Kong University of Science & Technology Ethics Committee.

## Results and Discussion

### Identification and Characterization of Internalized AChRs by using Quantum Dots

Because each streptavidin molecule contains 4 high-affinity binding sites for biotin, we sequentially labeled muscle cells with biotin-BTX and QD 655-conjugated streptavidin (hereafter referred to as BBQ) to induce AChR crosslinking on the cell surface. Instead of labeling AChRs with BBQ at low density to track single AChR molecules on the muscle surface [16], [17], in this study we applied BBQ at a high concentration to trigger AChR crosslinking and endocytosis.

**Figure 1:**
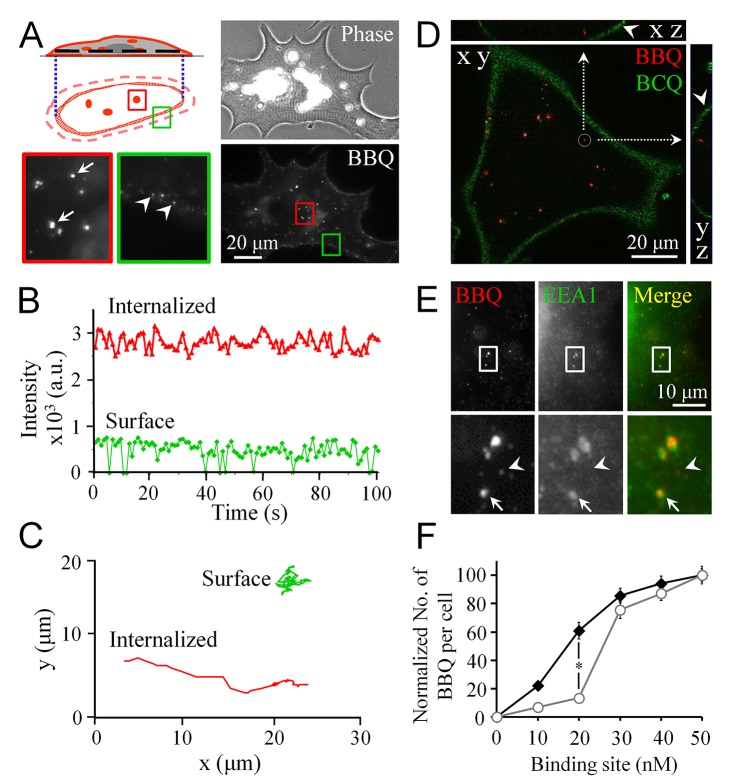
Induction of AChR endocytosis by crosslinking with biotin-BTX and QD-streptavidin (BBQ). **A**, A schematic diagram and representative images showing 2 pools of AChRs revealed by BBQ labeling. The fluorescence images were captured at the focal plane in the middle of a muscle cell (depicted as a black dotted line in the schematic diagram). Arrows indicate internalized AChRs (in the red box); arrowheads indicate surface AChRs (in the green boxes). The colored, boxed regions were magnified by 5 times for clarity. **B**, Two representative fluorescence intensity profiles of BBQ signals of distinguishing internalized and surface AChRs. Internalized AChRs (red curve) showed higher fluorescence intensity compared with surface AChRs (green curve). Moreover, because single QDs blink, the fluorescence intensity (background-subtracted) of the surface AChRs occasionally dropped to zero. **C**, Two representative trajectories showing distinct movement behaviors of internalized and surface AChRs. Linear movement represents internalized AChRs, whereas Brownian motion represents surface AChRs. **D**, A representative image reconstructed from a z-stack maximal projection showing the subcellular localization of BBQ-labeled internalized AChRs. To outline the cell membrane (arrowheads), the ganglioside GM1 was labeled with biotin-CTX and then with streptavidin-QD (labeled as BCQ). **E**, Colocalization of BBQ-labeled internalized AChRs with the early endosomal marker EEA1 (arrow). Arrowheads indicate newly internalized AChRs prior to endosomal fusion. **F**, Correlation between AChR binding sites and internalized AChRs. The concentration of AChR binding sites was manipulated by either reducing the QD-streptavidin concentration (filled circles) or by masking the biotin-binding sites of QD-streptavidin with free biotin (open circles). Data are mean ± SEM; *p<0.01 (Student’s *t* test).

Within 5 min after BBQ labeling, both surface and internalized pools of AChRs were detected when we observed the fluorescence signal at a focal plane near the center of the cells, as depicted in the schematic diagram in Fig. 1A. Two AChR pools could be readily distinguished based on the size, intensity, and trajectory of QD 655 signals. First, internalized AChRs were observed to be brighter and bigger in QD signals (Fig. 1A, arrows) when compared with the surface AChRs (Fig. 1A, arrowheads). Second, because of the intrinsic blinking property of individual QDs [22], the single QD-AChRs on the surface exhibited a weaker fluorescence signal that occasionally dropped to the background level (“blinking”). By contrast, the internalized AChRs were present within vesicles that each contained more than one QD-AChR, and these were detected as large packets that were substantially brighter than single surface BBQs; moreover, the fluorescence intensity in this case was stable and showed little fluctuation (Fig. 1B). Lastly, we determined that surface AChRs exhibited Brownian motion-like behavior, whereas the internalized AChRs exhibited almost linear movement (Fig. 1C), supporting the notion that the endocytic vesicles containing internalized AChRs are transported along cytoskeletal elements. This type of movement presumably leads the AChRs to lysosomal compartments for degradation, which results in the reduction of surface AChR density that is manifested in MG pathogenesis [23]. Thus, using this simple protocol involving BBQ labeling, we have developed an effective approach to induce AChR endocytosis that enables the behaviors of surface and internalized AChR pools to be visualized.

To further characterize the AChRs endocytosed through BBQ labeling, we examined the subcellular localization of the internalized AChRs. The cell surface was outlined by labeling for the ganglioside GM1 by using biotin-CTX, followed by QD 525-conjugated streptavidin. Unlike the BBQ labeling used for AChRs, this procedure did not result in the endocytosis of GM1 (data not shown). A reconstruction of z-stack images illustrated that internalized AChR vesicles were localized in cytosolic compartments away from the cell membrane (Fig. 1D, arrows). Following endocytosis, AChR-containing vesicles could fuse with the early endosomal compartment [24]. Immunostaining of an early endosomal marker EEA1 showed that most of the BBQ signals were colocalized with endosomal vesicles (Fig. 1E, arrows). By examining 254 puncta of BBQ fluorescence in 5 separate cells, we determined that 56.3% ±2.3% of the internalized AChR packets were colocalized with EEA1. We also detected certain BBQ-containing fluorescent bodies that were smaller than the aforementioned puncta and were not colocalized with EEA1 (Fig. 1E, arrowheads); these presumably were surface AChRs or newly internalized AChRs that had not fused with endosomes. At mature NMJs, the recycling of endocytosed AChRs back to the muscle surface has been identified using a well-established sequential-labeling protocol [25]. Because of the broad excitation spectra of the 2 kinds of QDs used in this study [26], we could not accurately determine the fractions of endocytosed AChRs in recycling versus lysosomal pathways by using the previously published sequential-labeling protocol that involves labeling with biotin-BTX and streptavidin-QD conjugates. Nevertheless, our results suggest that the AChRs that were endocytosed through crosslinking are targeted to endosomal compartments, which is similar to the fate of the AChRs whose internalization is induced by pathogenic anti-AChR antibodies in myasthenic patients [23].

In MG pathogenesis, antibody-induced AChR endocytosis is triggered by the crosslinking of multiple AChR molecules that are closely apposed to each other in the postsynaptic membrane [24]. To further confirm that QD-mediated AChR endocytosis was also mediated by receptor crosslinking, we examined the effects of altering the number of biotin-binding sites of the streptavidin conjugated to QDs. *Xenopus* muscle cells were labeled with biotin-BTX and subsequently streptavidin-QD labeling was performed using 2 methods: the first method involved serially diluting QD streptavidin, whereas the second involved pre-masking a fixed concentration of QD-streptavidin with various amounts of free biotin molecules. The molar concentration of AChR binding sites was calculated in each case, based on each QD being conjugated with a fixed number of streptavidin molecules (according to manufacturer’s specification) and on each streptavidin molecule featuring 4 biotin-binding sites. We found that the number of BBQ-labeled AChR vesicles was positively correlated with the molar concentration of AChR binding sites in both cases (Fig. 1F). However, at concentrations of binding sites lower than 30 nM, AChR endocytosis was triggered considerably more effectively by the QD-streptavidin prepared through serial dilution (filled symbols), whose biotin-binding sites were uncompromised, than by the QD-streptavidin prepared by partially masking the biotin-binding sites on streptavidin with free biotin (open symbols in Fig. 1F). Thus, of the 2 conjugates, the QD-streptavidin conjugates that retained full biotin-crosslinking ability more potently caused the endocytosis of biotin-BTX-labeled AChRs. These data further support the notion that BBQs mimic anti-AChR antibodies in triggering AChR endocytosis by crosslinking the receptors.

### Requirement of Clathrin and Microtubules in Crosslinking-induced Endocytosis of AChRs

**Figure 2:**
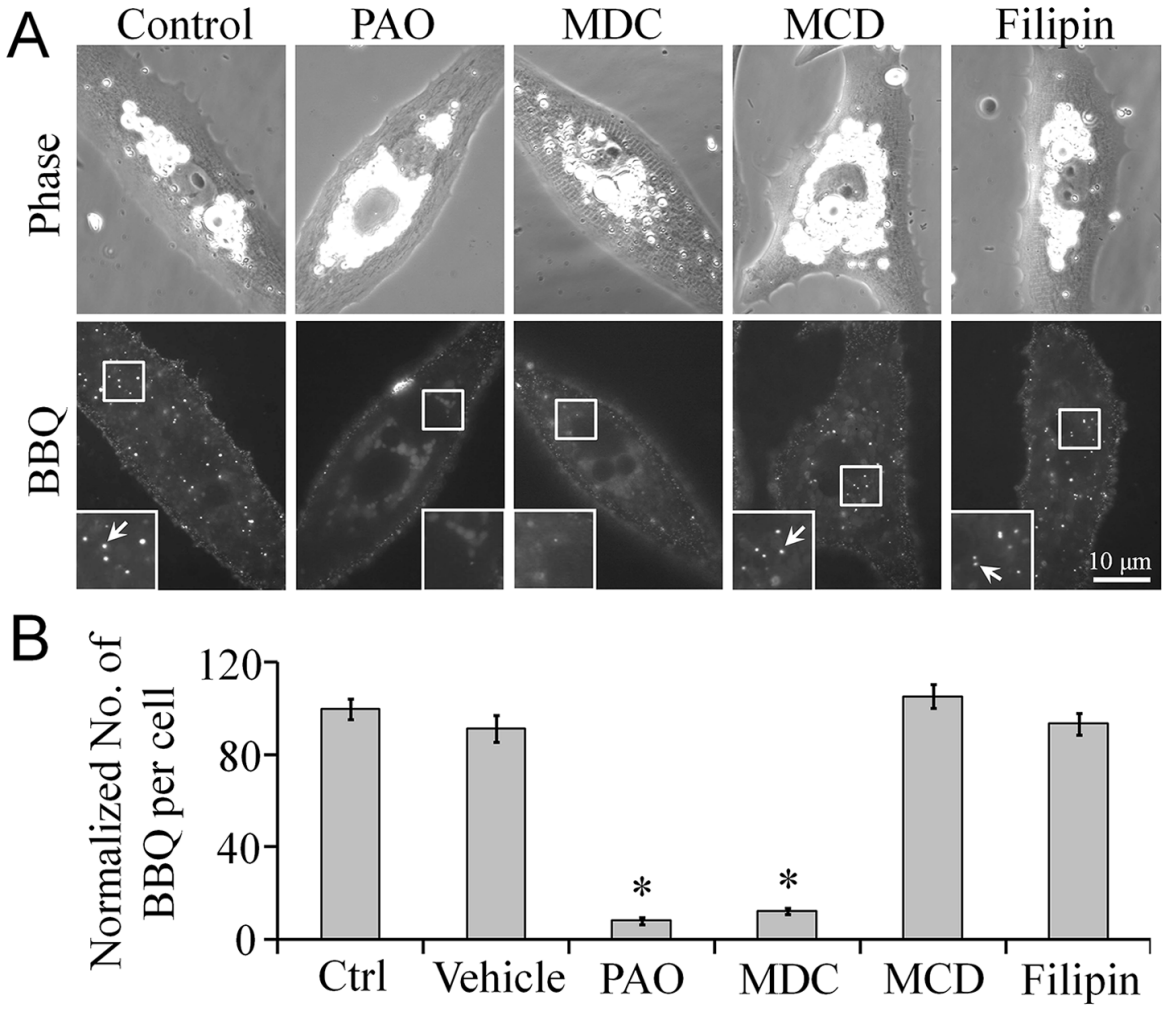
BBQ crosslinking-induced AChR endocytosis through clathrin-dependent, caveolin-independent mechanisms. **A**, Representative images showing the inhibition of crosslinking-induced AChR endocytosis by specific pharmacological inhibitors of clathrin (PAO and MDC) but not caveolin (MCD and filipin). For clarity, the boxed regions were magnified twofold, and the BBQ-labeled AChRs are marked with arrows in the insets. **B**, Quantification showing the effect of pharmacological agents on crosslinking-induced AChR endocytosis; n = 120, control; 90, vehicle, PAO, MDC, MCD, and filipin. Data are mean ± SEM; *p<0.001 (Student’s *t* test).

Clathrin- and caveolae-mediated endocytic mechanisms are 2 common pathways used for receptor internalization [27], [28]. Previously, in a muscle cell line, AChR internalization was shown to occur in the absence of clathrin or caveolin [24]. Here, we used a pharmacological approach to test whether clathrin and caveolin were required for AChR endocytosis induced by BBQ crosslinking. Cultured *Xenopus* primary muscle cells were treated with specific inhibitors of the clathrin- or caveolae-mediated pathway for 1 h before inducing AChR endocytosis through BBQ labeling. We found that 2 clathrin inhibitors, phenylarsine oxide (PAO; 10 µM) and monodansylcadaverine (MDC; 100 µM), used at concentrations that effectively inhibit the clathrin-mediated endocytic pathway [29], [30], potently blocked crosslinking-induced AChR endocytosis (Fig. 2). By contrast, treatment with 2 caveolae inhibitors, methyl-β-cyclodextrin (MβCD; 1 mM) [31] or filipin III (2.5 mg/L) [32], showed no effects when compared with the untreated control cells (Fig. 2). These results indicated that QD crosslinking-induced AChR endocytosis is regulated by the clathrin-mediated endocytic pathway in primary muscle cell cultures.

**Figure 3:**
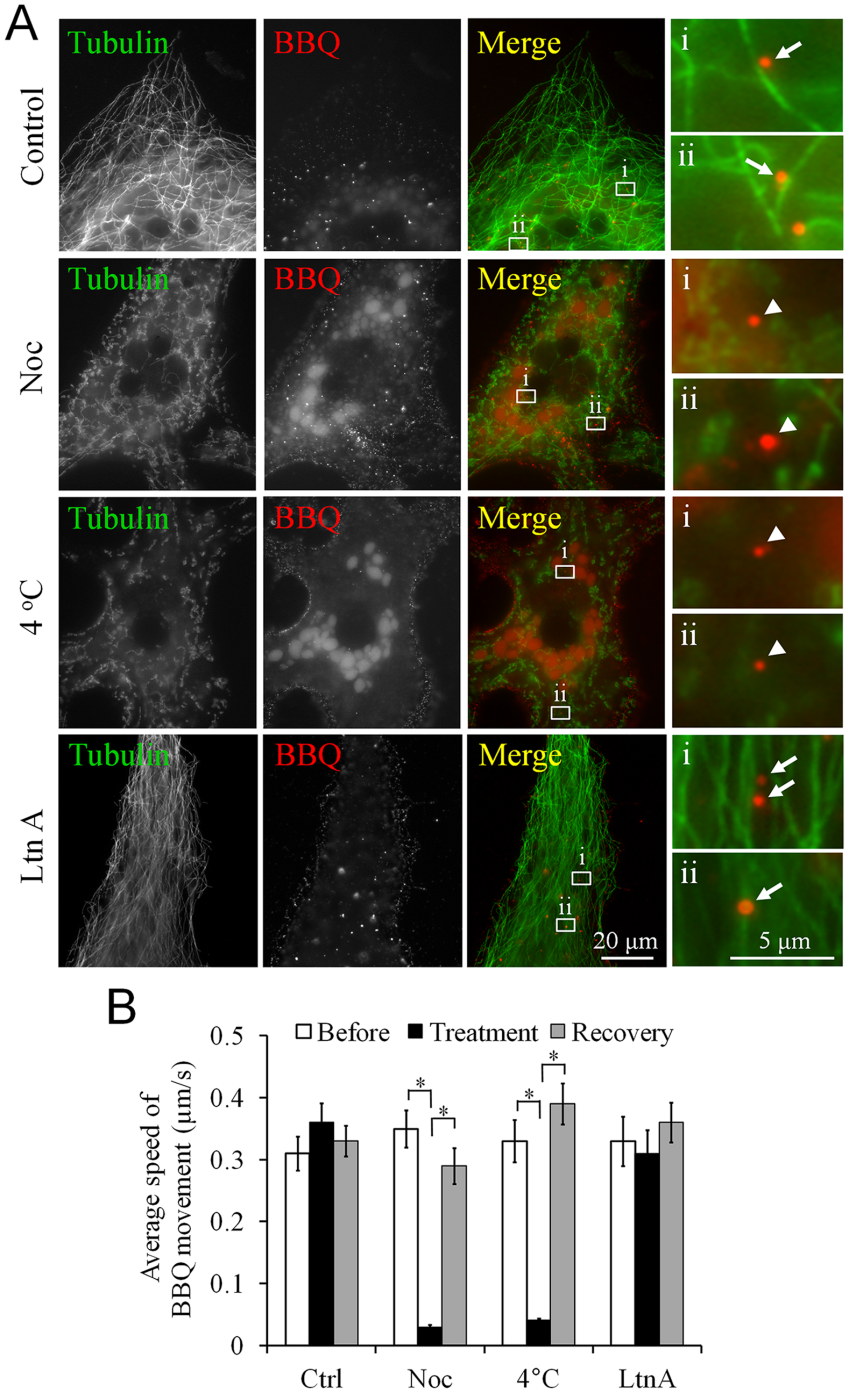
Microtubules, but not actin filaments, are required for intracellular movement of endocytosed AChR vesicles. **A**, Representative images showing the close association of BBQ crosslinking-induced AChR vesicles (BBQ) and microtubule structures (tubulin) in cultured muscle cells (arrows). The integrity of the microtubule network was effectively perturbed by treatment with nocodazole (Noc) or exposure to 4°C, which led to the fragmentation of the microtubule structures; however, microtubules were not affected by latrunculin A (Ltn A) treatment. Crosslinking-induced AChR endosomes were detected but were not associated with microtubule (arrowheads). The boxed regions were magnified by 8 times for clarity. **B**, Quantification showing the differential effects of cytoskeletal disruption of microtubules and actin filaments on the movement of crosslinking-induced AChR vesicles. Data are mean ± SEM; n = 30; *p<0.001 (Student’s *t* test).

After endocytosis, AChR vesicles/endosomes are thought to be transported on cytoskeletal tracks for short- and long-range movement, destined ultimately to fuse with lysosomal compartments where proteins are degraded [23]. Previous studies showed that whereas microtubules are not crucial for the early steps in the formation of endocytic vesicles [33], microtubule structures may be required for the long-range transport of AChR vesicles. Because internalized AChRs were transported almost linearly (Fig. 1C), we speculated that the trafficking of endocytosed AChR vesicles depended on the integrity of microtubule networks. In control muscle cells, most of the QD-labeled AChR vesicles were closely associated with microtubule structures, as revealed by tubulin immunostaining (Fig. 3A, arrows). Treating cultured muscle cells with the microtubule-depolymerizing agent nocodazole (at 1µM), or incubating the cells at 4°C, almost entirely disrupted the integrity of the microtubule network throughout the cells (Fig. 3A, arrowheads). In treated muscle cells, fragmented microtubule staining was consistently observed, in contrast to the long filamentous microtubule structures detected in control untreated muscle cultures. Next, we measured the velocity of endosomal movement before, during, and after the treatments. Quantifying data collected from 30 muscle cells showed that disrupting microtubules by using either nocodazole or cold treatment nearly eliminated the movement of endocytosed AChR vesicles (Fig. 3B). Importantly, the intracellular transport of AChR vesicles was restored when we replaced the nocodazole-containing medium with normal culture medium or incubated the cold-treated muscle cells back at 23°C for recovery (Fig. 3B). Unlike microtubule disruption, depolymerizing actin filaments by adding 50 µM latrunculin A (Ltn A) did not affect the integrity of the microtubule structures in muscle cells (Fig. 3A, bottom row) or the vesicular movement of endocytosed AChRs (Fig. 3B). These data suggest that microtubules play an essential role in the long-range intracellular transport of AChR vesicles, but that actin filaments may only be required for their short-range transport.

### Role of Agrin-MuSK Signaling in Crosslinking-induced AChR Endocytosis

**Figure 4:**
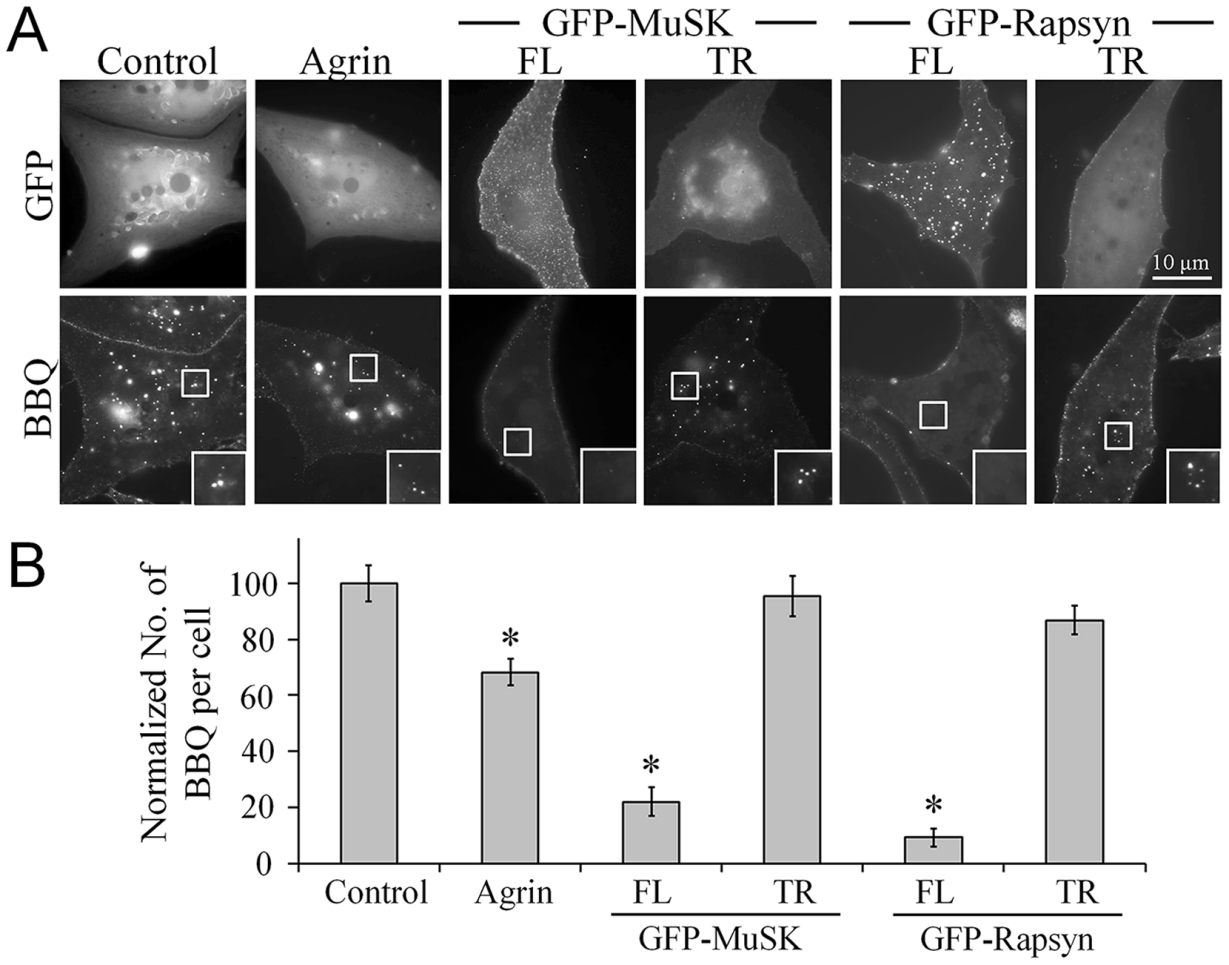
Inhibition of crosslinking-induced AChR endocytosis by agrin-MuSK signaling. **A**, Representative images showing the attenuation of AChR endocytosis by agrin-MuSK-rapsyn signaling. Muscle cells were either treated with agrin or distinct forms of GFP-MuSK or GFP-Rapsyn (full-length: FL; truncated form: TR) were overexpressed in muscle cells. For clarity, the boxed regions were magnified twofold and are shown in the insets. **B**, Quantitative analysis of the number of BBQ-labeled AChR vesicles formed in response to the activation of agrin signaling or the overexpression of its downstream molecules MuSK and rapsyn. Data are mean ± SEM; n = 172, control; 151, agrin; 97, GFP-MuSK FL; 101, GFP-MuSK TR; 65, GFP-Rapsyn FL; 98, GFP-Rapsyn TR; *p<0.001 (Student’s *t* test).

Crosslinking-induced AChR endocytosis triggered by BBQ labeling serves as a simple and reliable assay to investigate the cellular mechanisms underlying AChR endocytosis induced by pathogenic antibodies in MG. Previously, the turnover rate of AChRs at the synaptic sites (∼10 days) was demonstrated to be substantially lower than that at extrasynaptic regions (∼1 day) [34]–[36], indicating that the synaptic AChR clusters are considerably more stable than the diffuse extrasynaptic AChRs, and that nerve-derived signals may help stabilize synaptic AChRs and thereby prevent their internalization. The heparan-sulfate proteoglycan agrin is one such nerve-secreted signal that activates the muscle-specific kinase MuSK to induce AChR clustering at NMJs [37], [38]. Bath treatment of muscle cells with agrin reduces the degradation and turnover rate of AChRs [39]. In this study, we investigated the role of the agrin/MuSK signaling pathway in crosslinking-induced AChR endocytosis. Bath application of agrin markedly reduced the number of crosslinking-induced endocytosed AChR vesicles (by ∼30%) when compared with the level in control cells (Fig. 4B). Next, we overexpressed a full-length (FL) or a truncated mutant (TR) of MuSK in muscle cells and tested the crosslinking ability to trigger AChR endocytosis. Our results showed that MuSK-FL expression strongly inhibited AChR endocytosis induced by BBQ labeling, whereas the expression of MuSK-TR had no effect (Fig. 4). These data suggested a role of the agrin/MuSK signaling pathway in stabilizing the AChRs on the muscle surface and protecting them against endocytosis induced by crosslinking. Rapsyn is a key molecule that binds to AChR subunits and facilitates the vesicular transport of AChRs from the Golgi apparatus to the cell membrane after protein synthesis [40]. Rapsyn has been demonstrated to self-associate and mediate AChR cluster formation and to also link AChRs to the cytoskeleton [41], [42]; moreover, overexpression of rapsyn was shown to metabolically lower AChR turnover and protect the end-plate structures in muscle cells [10], [43], [44]. In our experiment, we tested the function of rapsyn in crosslinking-induced AChR endocytosis. Overexpression of GFP-tagged full-length (FL) rapsyn markedly reduced the number of endocytosed AChR vesicles induced by BBQ crosslinking, as compared with control cells expressing GFP alone (Fig. 4). By contrast, the truncated (TR) rapsyn mutant, which lacked the coiled-coil domain (Δ297–331) required for binding to AChR β subunit [42], showed no effect on crosslinking-induced AChR endocytosis (Fig. 4). Taken together, these results suggest that activation of the agrin-MuSK signaling pathway can protect diffuse AChRs against endocytosis induced by QD crosslinking.

### Potentiation of Crosslinking-induced Dispersal of Aneural AChR Clusters by Synaptogenic Stimulation

**Figure 5:**
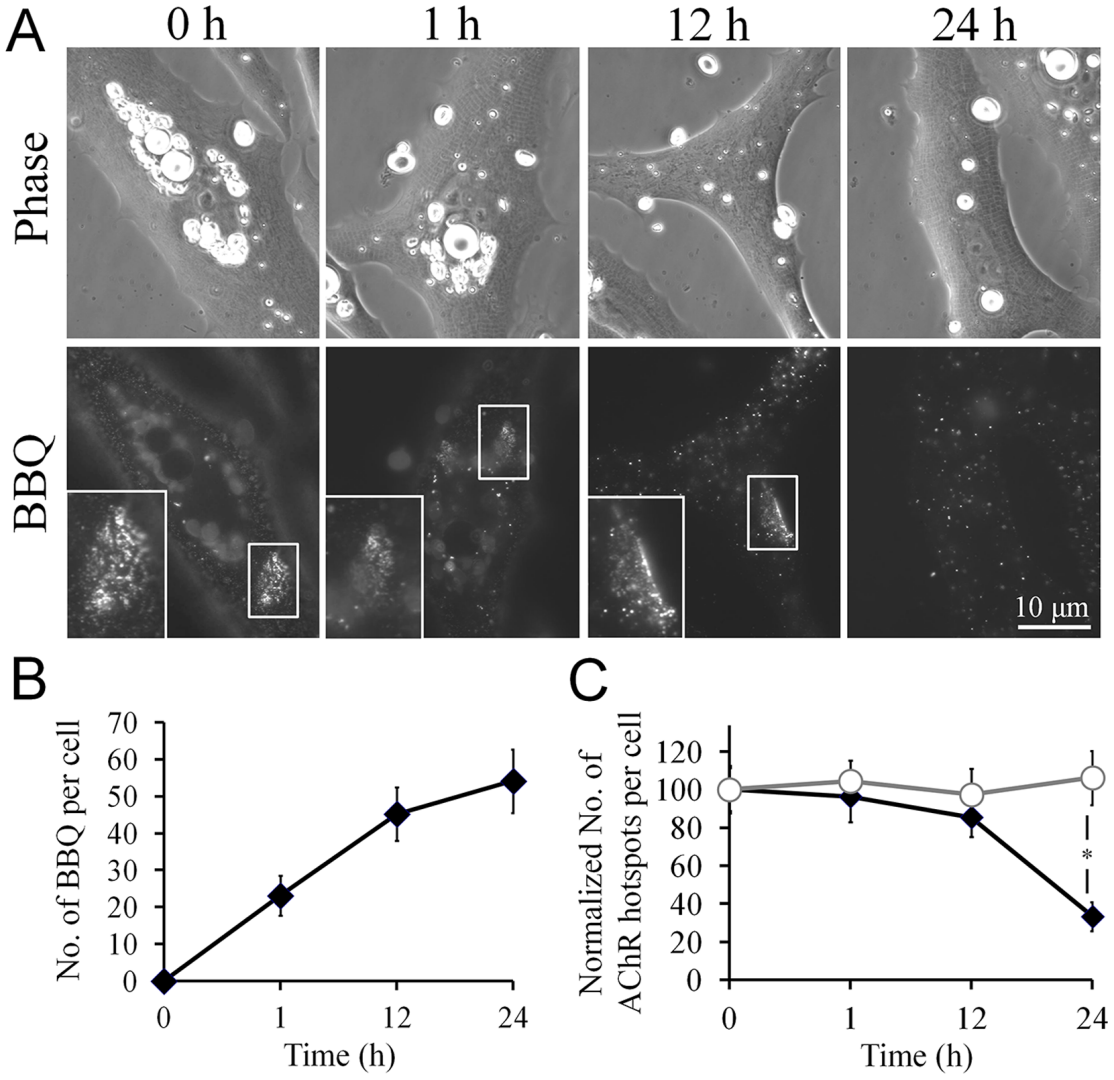
Dispersal of AChRs in aneural clusters triggered by crosslinking-induced endocytosis. **A**, Representative images showing the presence of spontaneously formed aneural AChR clusters at various time points in cells in which AChR endocytosis was induced by crosslinking. Cultured *Xenopus* muscle cells were labeled with BBQ at 0 h and the dispersal of aneural AChR clusters was followed over a course of 24 h. For clarity, aneural AChR clusters, where present, are indicated by boxes and are magnified in the insets. **B**, Quantification showing the time course of BBQ crosslinking-induced formation of AChR vesicles; n = 86. **C**, Quantification showing the number of aneural AChR clusters in relation to time in cultured muscle cells with (filled marks) or without (open marks) BBQ crosslinking. Data are mean ± SEM; n = 101, control; 95, with BBQ crosslinking; *p<0.001 (Student’s *t* test).

Before innervation, AChR clusters form spontaneously in the muscle membrane *in vitro* and *in vivo* through a process that is independent of the presence of agrin or other nerve-derived signals [45], [46]. Whether the AChRs that were internalized by BBQ labeling were derived from diffuse or clustered AChRs was unclear. Therefore, we investigated the effect of crosslinking-induced AChR endocytosis on the stability of aneural AChR clusters. We labeled *Xenopus* muscle cells with BBQ to induce AChR endocytosis and then monitored the cells at 1, 12, and 24 h after labeling. We detected an increase in the number of BBQ-labeled AChR vesicles over the course of 24 h (Fig. 5A and B). However, the number of aneural AChR clusters per cell remained unchanged for up to 12 h after BBQ crosslinking (Fig. 5C, black curve), and the disappearance of aneural AChR clusters became noticeable only at 24 h after BBQ crosslinking. These data suggest that the BBQ-labeled AChR vesicles detected at early time points are likely derived from the crosslinking and endocytosis of diffuse AChRs in the cell surface and, furthermore, that the AChR molecules present within aneural clusters are more resistant to crosslinking-induced endocytosis than diffuse receptors.

**Figure 6:**
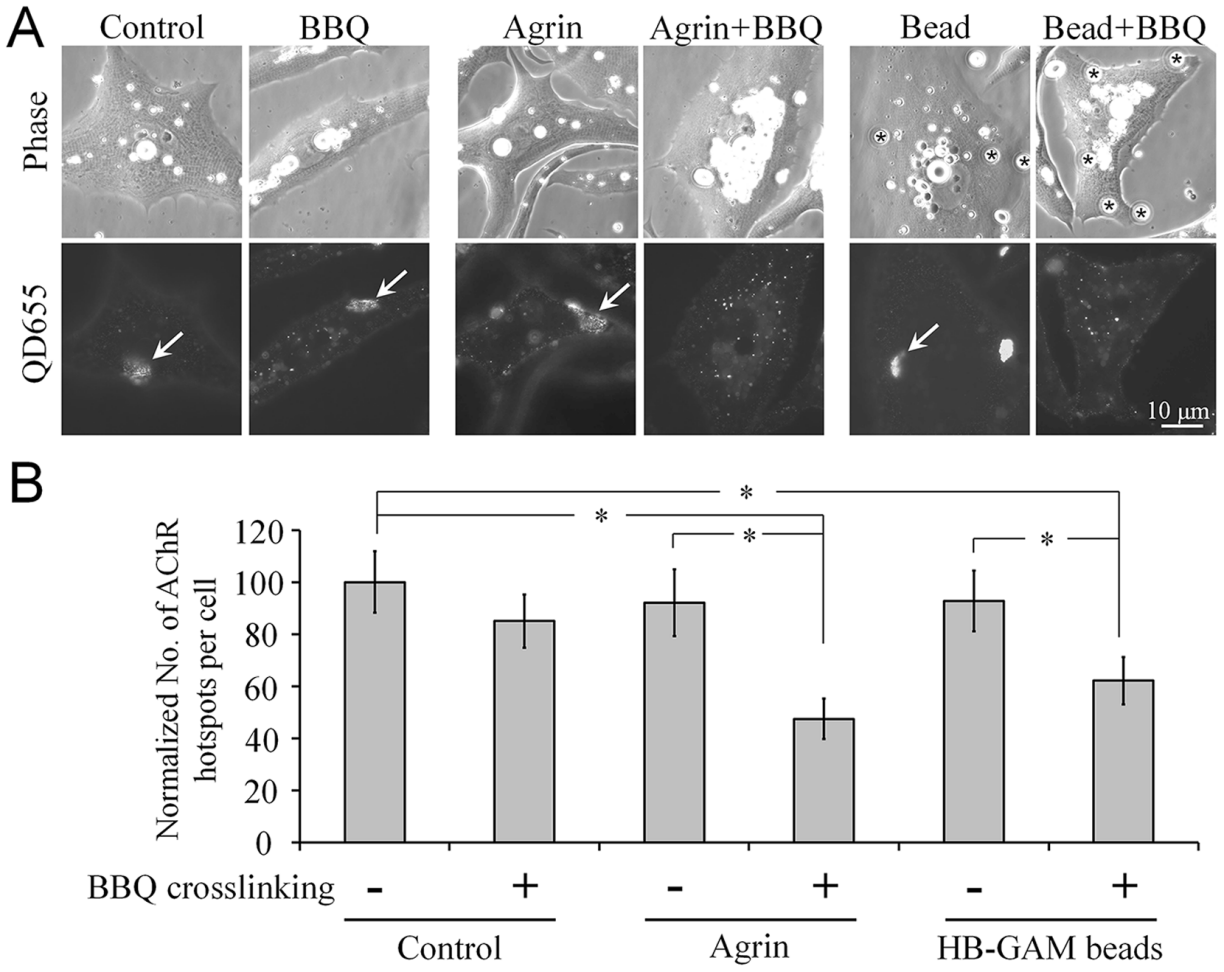
Enhancement of crosslinking-induced dispersal of aneural AChR clusters by synaptogenic stimulation. **A**, Representative images showing the effects of synaptogenic signals on the dispersal of aneural AChR clusters triggered by BBQ crosslinking. The aneural AChR clusters (arrows) were counted in cultured muscle cells in the presence or absence of BBQ crosslinking, in combination with agrin treatment (middle 2 columns) or HB-GAM bead stimulation (last 2 columns). **B**, Quantification showing the synergistic action of AChR crosslinking and synaptogenic signaling on the dispersal of aneural AChR clusters. Data are mean ± SEM; *p<0.01 (Student’s *t* test).

Although the aneural AChR clusters were determined to be more stable than diffuse AChRs, synaptogenic signals could conceivably redistribute AChRs from aneural to synaptic sites during synapse formation [8], [47]. We speculated that under synaptogenic stimulation, the structural scaffold that stabilizes surface AChRs at the aneural clusters may be compromised, thus making the receptors highly susceptible to crosslinking-mediated endocytosis. Previously, MuSK-containing synaptic components were suggested to be stabilized by the actin cytoskeletal scaffold through the interaction of the cytoplasmic protein rapsyn and α-actinin during AChR clustering [9], [48]. To test this possibility, we stimulated cultured muscle cells with 2 types of synaptogenic signals. Muscle cells were treated either globally with agrin or locally with latex beads coated with HB-GAM; treating muscle cells with agrin and HB-GAM beads effectively induces global and focal clustering of AChRs, respectively [37], [49]. Moreover, the presentation of synaptogenic signals induces the dispersal of aneural AChR clusters located at extrasynaptic sites [47], [50], [51]. We hypothesized that synaptogenic signals and AChR crosslinking synergize in causing the destabilization of aneural AChR clusters and lead to the dispersal of these clusters upon synaptic induction. Here, BBQ labeling of cultured muscle cells for a prolonged period (12 h) did not strongly affect the number of aneural AChR clusters present in muscle cells (Fig. 6), consistent with the results shown in Fig. 5C. Similarly, presenting synaptogenic signals alone (treatment with agrin for 4 h or HB-GAM beads for 6 h) did not strongly influence the stability of aneural AChR clusters during the time frame of their application. However, when we combined BBQ labeling with the presentation of synaptogenic signals, the disappearance of aneural AChR clusters was accelerated. These results suggest that synaptogenic signals weaken the association between surface AChRs and the cytoskeletal scaffold at the aneural clusters, which may facilitate crosslinking-induced endocytosis and lead to the disappearance of aneural AChR clusters.

In summary, we have presented a novel approach to study the mechanisms by which AChRs are trafficked upon receptor crosslinking and endocytosis triggered by BBQ labeling. These mechanisms mimic those that mediate AChR endocytosis induced by pathogenic anti-AChR antibodies in MG patients. We have further provided evidence supporting the view that crosslinking-induced AChR endocytosis involves clathrin-mediated and microtubule-dependent mechanisms. Moreover, synaptogenic signals were shown to stabilize diffuse AChRs globally in the muscle surface and protect them against crosslinking-induced endocytosis, and, conversely, to also potentiate crosslinking-induced dispersal of aneural clusters at extrasynaptic regions of muscle. Our previous studies have suggested that during synapse formation, the activation of the tyrosine phosphatase Shp2 by synaptogenic stimulation acts globally to destabilize aneural AChR clusters by countering the action of receptor tyrosine kinases [51]–[53]. Whether Shp2 is also involved in propagating local synaptogenic signals to mediate the global protection of diffuse AChRs against crosslinking-induced endocytosis remains to be determined. Elucidating how AChRs are stabilized and protected against crosslinking-induced endocytosis may lead to a new therapeutic method of MG prevention. In this study, we developed a simple and reliable assay to investigate the cellular and molecular mechanisms underlying AChR endocytosis and endocytic trafficking in neuromuscular development and diseases. Our results may also provide insights into potential therapeutic interventions that could be used to modulate the AChR endocytic pathway during MG pathogenesis.
